# Supplementary material for: Recent Developments in the Biological Activities, Bioproduction, and Applications of Pseudomonas spp. Phenazines
Source: Molecules. 2023 Feb 1;28(3):1368. doi: 10.3390/molecules28031368 (PMC9919295; doi:10.3390/molecules28031368)
Supplement: Supplementary file 1 [file molecules-28-01368-s001.zip › molecules-2057393-supplementary.pdf]

Supplementary materials

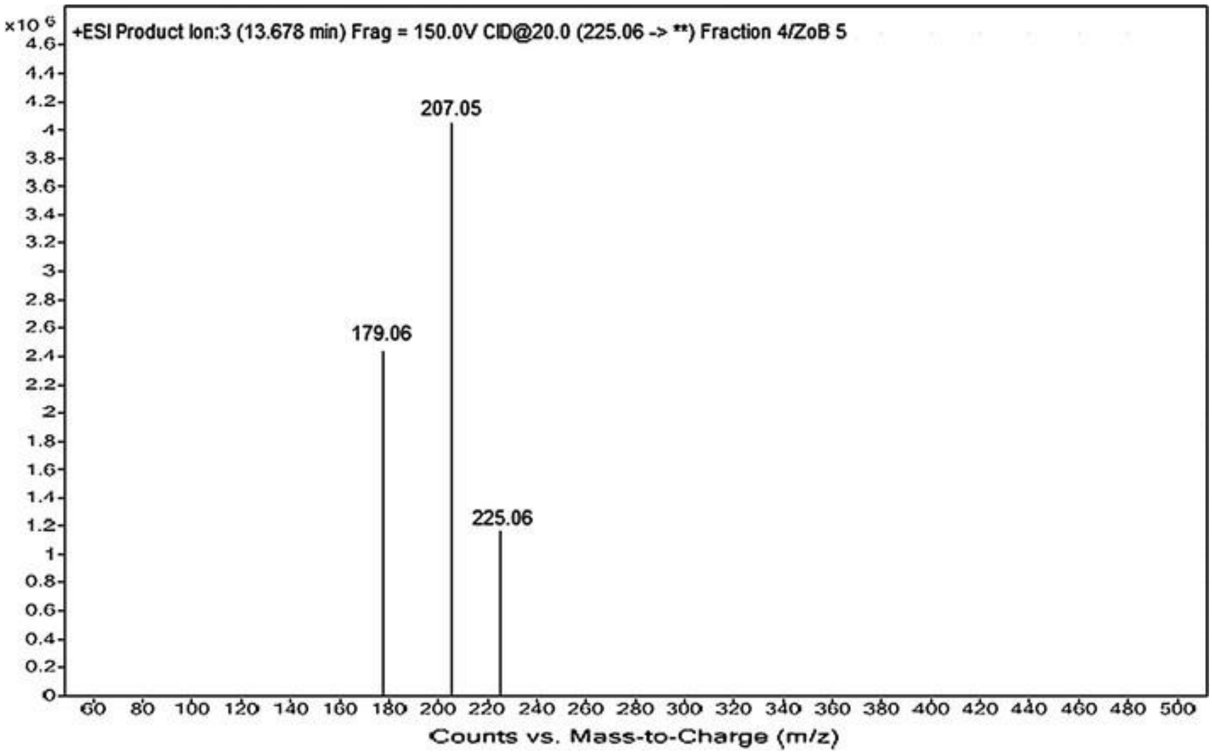

Figure S1. Mass spectrum of purified active fraction using LC_MS analysis [71].

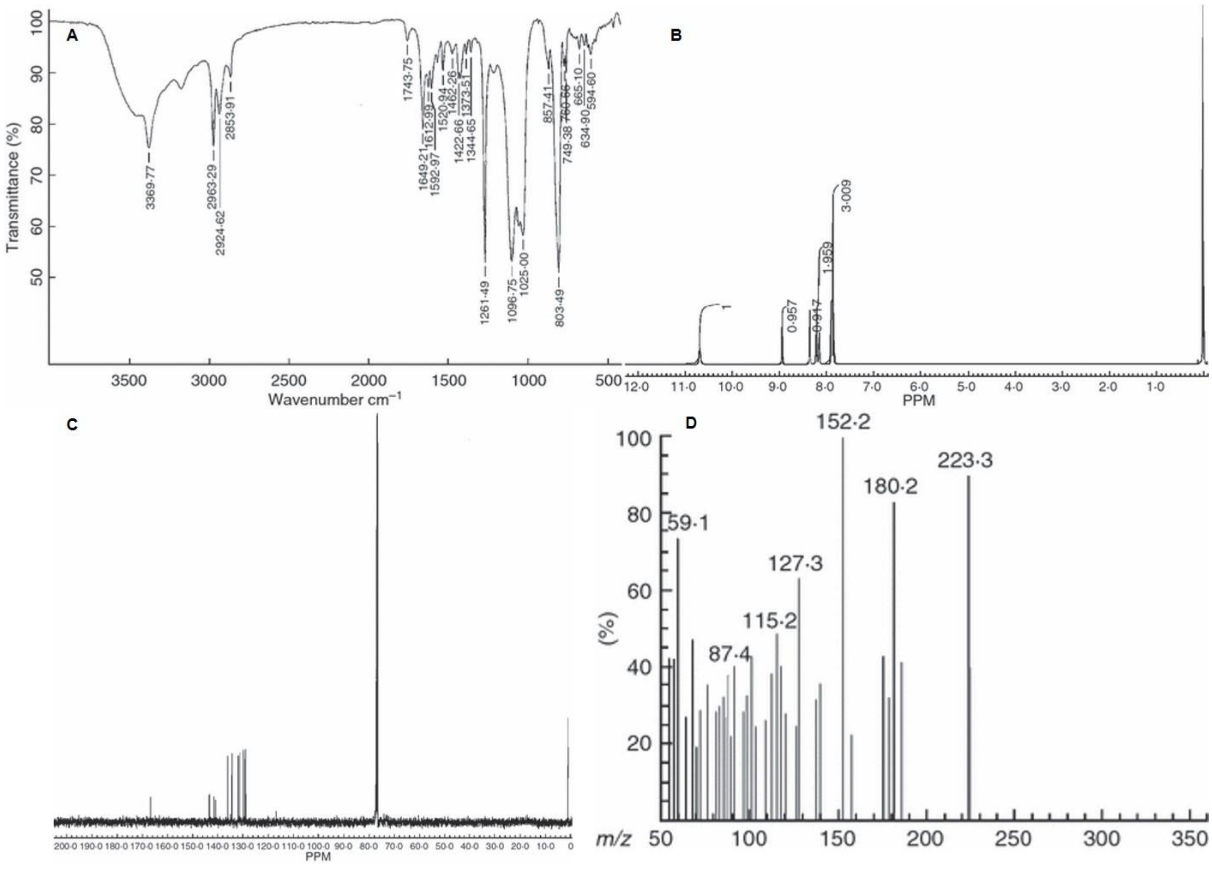

Figure S2. Characterization and identification of the purified compound of *P. aeruginosa* MML2212 A) IR spectrum; B) ^{1}H NMR; C) ^{13}C NMR and D) Mass spectrum.

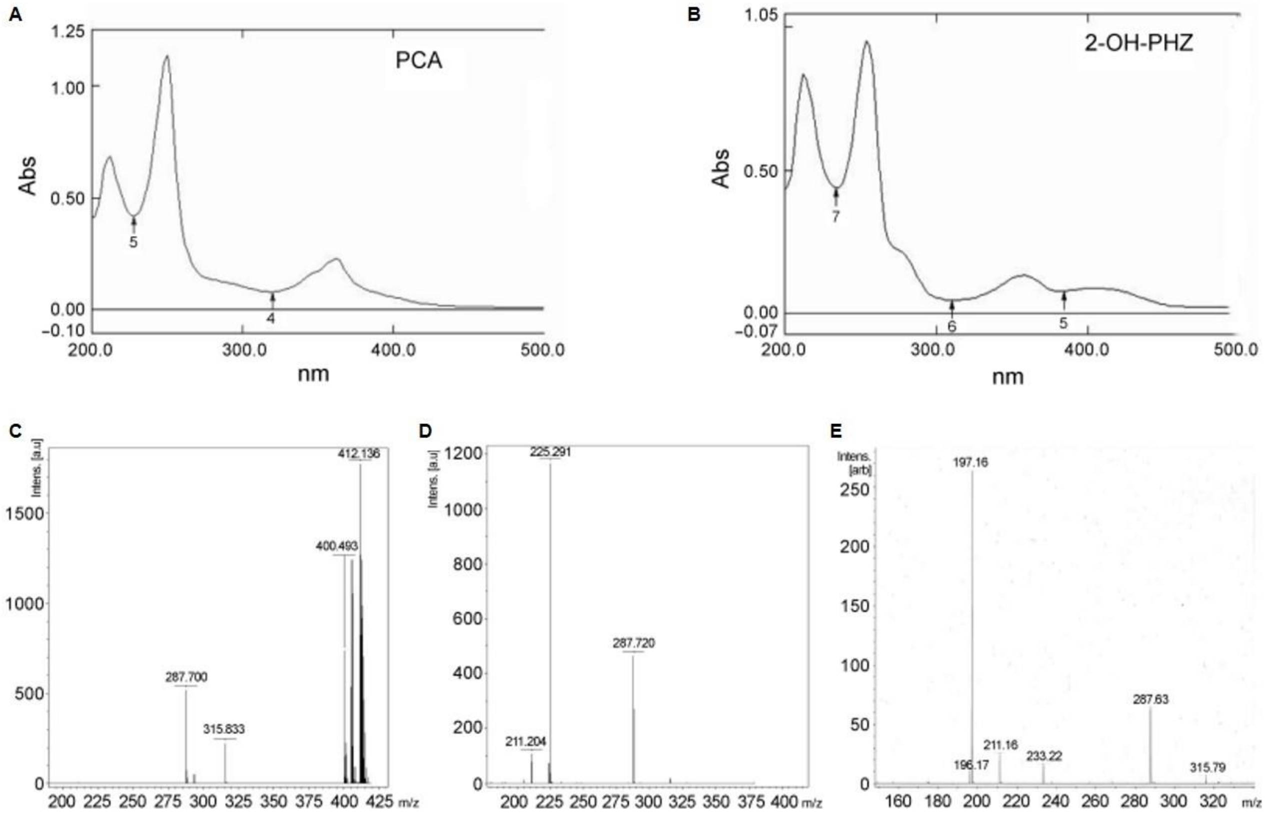

Figure S3. Quantification and identification of PCA and 2-hydroxyphenazine (2-OH-PHZ) from *P. aurantiac*a PB-St2. A) UV spectra of PCA and B) 2-OH-PHZ; C) Mass spectrum of “matrix A”; D) Mass spectrum of “matrix A” spiked with PCA, E) Mass spectrum of “matrix A” spiked with 2- OH-PHZ.
